# Leakage-Resistant Evaluation of Gait Mat and Multisensor Biomechanical Features for Knee Osteoarthritis Screening: A Subject-Level Data Integrity Study

**DOI:** 10.3390/bioengineering13090965

**Published:** 2026-08-24

**Authors:** Mi-Ae Yang, Kang-Su Ha

**Affiliations:** 1 Department of Science and Technology Convergence, Graduate School, Chosun University, Gwangju 61452, Republic of Korea; 2 Department of Psychiatry, Rainbow Hospital, Gwangju 61924, Republic of Korea; 3 College of Medicine, Chosun University, Gwangju 61452, Republic of Korea

**Keywords:** knee osteoarthritis, gait biomechanics, instrumented gait mat, smart insole, inertial measurement unit, multisensor fusion, biomedical engineering, machine learning, data integrity, internal evaluation

## Abstract

Selecting a sensing architecture for knee osteoarthritis (OA) screening requires balancing biomechanical information, system complexity, and reproducibility. We audited a public Korean multimodal gait dataset and performed a leakage-resistant internal evaluation. The release contained 180 participants (90 normal, 90 knee OA) measured using a smart insole, instrumented gait mat, and inertial measurement units (IMUs); all 1080 JavaScript Object Notation (JSON) files were checked for structural, value, provenance, and duplication errors. The primary benchmark was a fixed class-balanced L2 logistic regression model using nine gait mat variables, evaluated with subject-level repeated stratified five-fold cross-validation and 10,000 outcome-stratified bootstrap resamples. The audit identified 14 source-path metadata errors and one opposing-label duplicate smart insole payload, but no parsing, schema, range, or cross-partition subject errors. The gait mat model achieved an area under the receiver operating characteristic curve (AUROC) of 0.924 (95% confidence interval [CI], 0.879–0.962), balanced accuracy 0.883 (0.833–0.928), sensitivity 0.856, specificity 0.911, and Brier score 0.102. Adding smart insole and/or IMU features did not improve AUROC. Provider-model reproduction was descriptive because the public Validation partition informed model selection. In this release, the compact gait mat feature set provided the most favorable observed balance of discrimination, interpretability, and sensing complexity; external prospective evaluation is required before clinical use.

## 1. Introduction

Knee osteoarthritis is a common chronic joint disorder associated with pain, mobility limitation, and substantial disability. Its clinical manifestations include changes in walking speed, step length, stance and swing timing, joint motion, and load distribution [1,2,3,4]. Objective gait assessment can therefore complement symptoms and imaging by quantifying functional alterations that are not fully captured by static examinations.

Instrumented gait mats provide reproducible spatiotemporal measurements such as velocity, step length, stance time, swing time, and double support time [5,6]. Wearable smart insoles and inertial measurement units (IMUs) extend assessment beyond the laboratory and have supported machine learning approaches for identifying knee arthropathy and osteoarthritis-related gait signatures [3,7,8]. From a bioengineering perspective, however, each added modality also introduces hardware, calibration, synchronization, preprocessing, and data management requirements. Multisensor fusion is therefore most useful when the added modality contributes nonredundant biomechanical information sufficient to justify the extra system complexity.

Prediction model performance can also be overstated by data leakage, tuning and evaluation on the same partition, non-independent repeated measurements, or post hoc selection of the best-performing configuration. Transparent reporting and subject-level resampling are especially important when multiple files or sensor streams originate from the same participant [9,10,11]. Discrimination alone is insufficient; calibration and overall probabilistic accuracy should also be examined [12,13]. Moreover, favorable internal results do not establish transportability to new clinical settings [14,15].

The AI Hub Musculoskeletal Disease Biosignal dataset was constructed using smart insole, gait mat, and IMU measurements from normal and knee osteoarthritis groups [16]. Its documentation describes 200 participants and an 80:10:10 training–validation–test split, but the public download contained only the training and validation partitions. The release also provides source code and pretrained weights for three provider models. This combination provides an opportunity to examine a practical bioengineering question: how much discriminatory information is retained by each sensing configuration, and whether additional modalities add value beyond a simpler gait mat platform when data integrity and subject-level leakage are explicitly controlled.

This study had four objectives: (1) to perform a subject-level structural and value-level audit of the public release; (2) to establish a leakage-resistant internal biomechanical benchmark using a fixed, interpretable gait mat logistic regression model with outcome-stratified bootstrap confidence intervals; (3) to quantify the incremental value of smart insole and IMU features relative to the additional sensing and data complexity they introduce; and (4) to reproduce the provider-supplied models as an explicitly descriptive secondary analysis. We did not claim external validation or clinical diagnostic readiness.

## 2. Materials and Methods

### 2.1. Study Design and Reporting

This was a secondary analysis of a publicly released, deidentified sensor dataset and accompanying model files. The study combined a data integrity audit with a bioengineering evaluation of alternative sensing configurations for prediction model development and internal performance assessment. Reporting was guided by the Transparent Reporting of a multivariable prediction model for Individual Prognosis Or Diagnosis plus Artificial Intelligence (TRIPOD + AI) statement [9]. To avoid ambiguity, the term evaluation is used for performance assessment; internal evaluation denotes resampling within the available public cohort.

The final analysis plan was frozen on 31 July 2026, before calculation of the final 10,000-resample bootstrap confidence intervals and manuscript-level interpretation. It was not prospectively registered and followed preliminary exploratory comparisons that informed selection of the gait mat primary model. Consequently, the reported confidence intervals quantify uncertainty conditional on the frozen out-of-fold predictions and do not account for the earlier model-selection process.

### 2.2. Data Source, Participants, and Public Release

The source was the AI Hub “Musculoskeletal Disease Biosignal Data” dataset (dataset no. 71966; version 1.1), constructed in 2025 under the supervision of Seoul National University Hospital [16]. The documentation describes 100 normal participants and 100 participants with knee osteoarthritis. Each participant was measured with a smart insole, an instrumented gait mat, and an IMU system. Smart insole data were collected over 15 days in daily life, with 10 days selected for the released structured dataset; gait mat and IMU measurements were obtained under supervised walking protocols.

The public download contained 160 participants in the Training partition (80 per class) and 20 in the Validation partition (10 per class), for a total of 180 publicly accessible participants. The 20-subject Test partition described in the documentation was not present in the public file list and was not available after direct confirmation. We therefore combined the public Training and Validation partitions for the primary repeated cross-validation analysis. The original partition labels were retained for audit and for the separate reproduction of provider-supplied pretrained models. Participant characteristics for the publicly released cohort are summarized in Table 1.

### 2.3. File Structure and Data Integrity Audit

The public release comprised 1080 JavaScript Object Notation (JSON) files: 540 source data files and 540 labeling files across three sensors and 180 participants. For every labeling file, a deterministic parser extracted the participant identifier, class label, sensor values, and recorded source path. The audit evaluated: JSON parse success; required keys and schema; one-to-one raw label file correspondence; exact equality of sensor values between source and labeling files; numeric convertibility; documentation-defined ranges; smart insole day counts; duplicate measurement payloads; participant overlap between public partitions; cross-sensor participant and class consistency; and agreement between recorded source paths, label-derived filenames, and class folders.

Duplicate sensor payloads were identified by hashing canonically ordered JSON value objects. A duplicate with opposing class labels was treated as a high-priority integrity finding. Metadata path errors were distinguished from sensor value errors: a path defect was recorded even when the labeling file contained values identical to the correctly matched source file.

### 2.4. Outcome and Predictor Sets

The binary outcome was the provider label: 0 for normal and 1 for knee osteoarthritis. The dataset did not provide radiographic Kellgren–Lawrence grade, symptom severity, treatment status, or an independently adjudicated outcome for this secondary analysis.

The primary predictor set contained all nine gait mat variables: left and right step length, velocity, left and right stance phase percentage, left and right swing-phase percentage, and left and right double-support time. The fixed secondary predictor sets were: smart insole alone (14 variables per day across 10 days; 140 features), IMU alone (eight bilateral kinematic variables), smart insole plus gait mat, smart insole plus IMU, gait mat plus IMU, and all three sensors. Demographics, participant identifiers, source paths, diagnosis text, symptoms, disease side, region, and class labels were excluded from all model inputs.

### 2.5. Frozen Primary Model and Subject-Level Internal Evaluation

The frozen primary model was class-balanced L2-penalized logistic regression with C = 0.3, the liblinear solver, and a probability threshold of 0.50. Median imputation and z-score standardization were estimated only from the training portion of each fold and then applied to the held-out fold. No feature selection, hyperparameter tuning, or threshold optimization was performed during the final analysis.

Internal evaluation used repeated stratified five-fold cross-validation with 10 repeats and random seed 20260731. The unit of splitting was the participant; all sensor measurements from a participant remained together. Each participant generated one out-of-fold probability in every repeat. The 10 out-of-fold probabilities were averaged at the participant level before the final performance metrics and bootstrap intervals were calculated. This design prevents repeated smart insole days and cross-sensor records from the same participant from entering both the training and evaluation portions of a fold.

### 2.6. Secondary Sensor-Configuration Analyses

Each secondary sensor configuration used the same fixed logistic regression specification, fold assignments, preprocessing rules, threshold, and participant-level probability averaging as the primary analysis. Paired differences in area under the receiver operating characteristic curve (AUROC) for configurations that contained the gait mat were estimated against the primary gait mat model by applying the same outcome-stratified bootstrap samples to both models. A sensitivity analysis excluded the two participants involved in the opposing-label smart insole duplicate.

### 2.7. Reproduction of Provider-Supplied Pretrained Models

The provider package contained Python source code and pretrained weights for a smart insole model, a smart insole plus gait mat model, and a smart insole plus IMU model. Code and state dictionaries were structurally inspected and loaded without modification. The supplied implementations used sensor values only; no direct target-text or demographic leakage was identified. Training partition medians and scalers were refitted from the 160 public Training participants and applied to the 20 public Validation participants before inference with the supplied weights.

These reproduction results were designated descriptive secondary analyses. The provider code used the public Validation partition for early stopping and best-weight selection, and it also referenced a separate Test path that was absent from the public release. Therefore, performance on the public Validation partition was neither an independent test nor an external evaluation.

### 2.8. Performance Measures and Statistical Analysis

The co-primary performance measures were the area under the receiver operating characteristic curve (AUROC) and balanced accuracy. Secondary measures were area under the precision-recall curve (AUPRC), accuracy, F1 score, sensitivity, specificity, positive predictive value (precision), Brier score, calibration intercept, and calibration slope [12,13,17]. Classification measures used the frozen threshold of 0.50. Calibration intercept and slope were obtained by regressing the outcome on the logit of the participant-level predicted probability.

Uncertainty was quantified with 10,000 nonparametric bootstrap resamples stratified by outcome class. Within each resample, 90 normal and 90 knee osteoarthritis participants were sampled with replacement from the participant-level mean out-of-fold predictions. Percentile 95% confidence intervals were reported. For paired AUROC comparisons, the same bootstrap indices were applied to the primary and comparator predictions. The bootstrap procedure did not refit the complete cross-validation pipeline and therefore represents conditional uncertainty around the frozen participant-level predictions rather than the full variability of model development.

Analyses were conducted in Python 3.13.5 using NumPy 2.3.5, pandas 2.2.3, scikit-learn 1.8.0, and Matplotlib 3.10.8 [18]. Provider weights were reproduced on CPU using PyTorch 2.10.0; the provider documentation specified PyTorch 2.3.1. OpenAI ChatGPT (GPT-5.6 Sol, web interface, accessed July–August 2026) was used to assist with drafting and reviewing data audit and statistical analysis scripts, organizing the analytical workflow, preparing tables and figures, structuring the manuscript, and improving language and formatting. All source data checks, code execution, numerical results, tables, figures, references, methodological decisions, and interpretations were independently reviewed and verified by the authors.

### 2.9. Ethics

The dataset documentation states that the original collection received institutional review board approval and participant consent at the participating hospital and that released records were deidentified. The present study involved secondary analysis of publicly accessible deidentified files, no participant contact, and no access to direct identifiers; additional informed consent and new institutional review board review were not applicable to this analysis.

**Figure 1:**
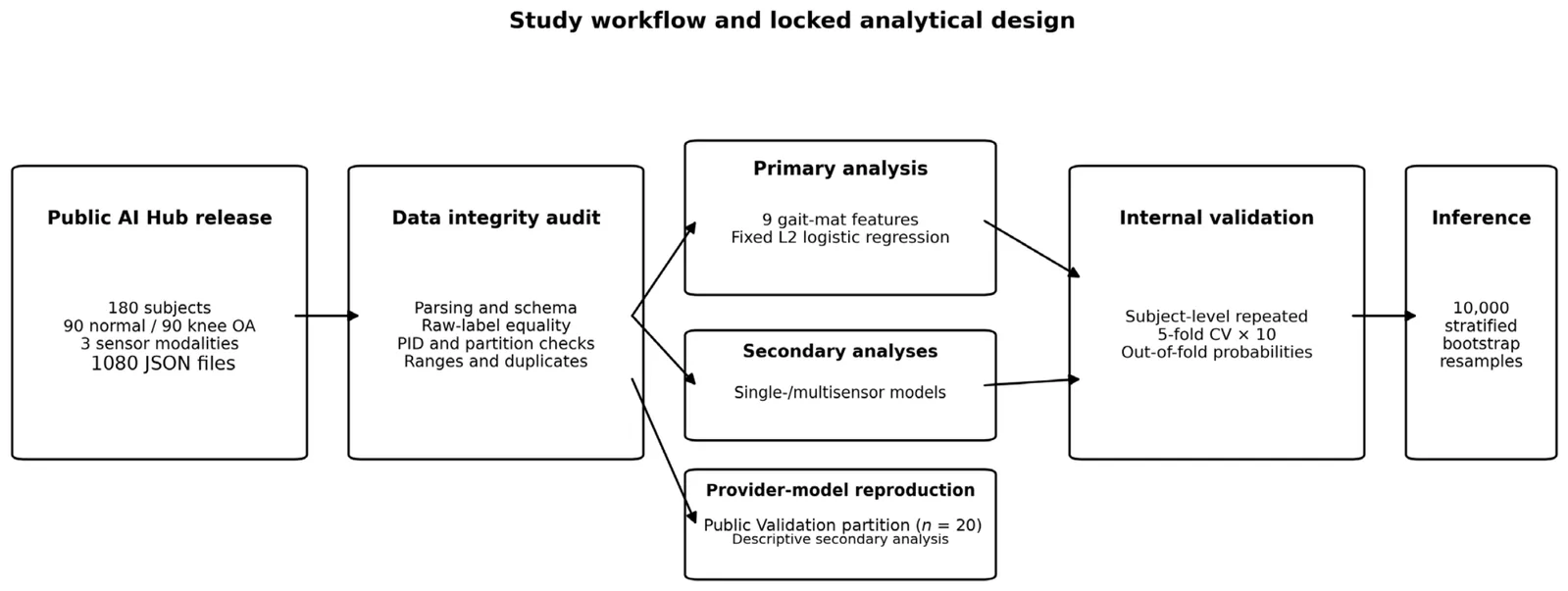
Study workflow. The public Training and Validation partitions were combined only for the primary subject-level repeated cross-validation analysis. Provider model reproduction retained the original public partitioning. CV, cross-validation; JSON, JavaScript Object Notation; OA, osteoarthritis; PID, participant identifier.

The complete study workflow is shown in Figure 1.

## 3. Results

### 3.1. Public Cohort and Data Integrity Audit

The public cohort included 180 participants, evenly divided between normal and knee osteoarthritis labels. The knee osteoarthritis group was older and included a higher proportion of women than the normal group (Table 1). Among participants with knee osteoarthritis, 70 had bilateral, 12 right-sided, and 8 left-sided disease labels.

All 1080 JSON files were parsed successfully. Every sensor had 160 unique Training participants and 20 unique Validation participants, with complete participant intersection across the three sensors and no overlap between public partitions. No required-key or schema errors, raw-label value mismatches, numeric conversion failures, documentation-range violations, or smart insole day count errors were found.

Fourteen labeling files contained inaccurate source-path metadata: 11 gait mat and 3 IMU files. These were path-string defects; the sensor values in the labeling files matched their correctly paired source files. One smart insole measurement payload was duplicated exactly across two Training participants with conflicting labels (SUBJ_247, normal; SUBJ_253, knee osteoarthritis). The main gait mat predictor values for these participants were not identical. The complete data integrity audit is summarized in Table 2.

### 3.2. Primary Gait Mat Model

The frozen nine-variable gait mat logistic regression model achieved an AUROC of 0.924 (95% CI, 0.879–0.962) and balanced accuracy of 0.883 (95% CI, 0.833–0.928). At the prespecified 0.50 threshold, the confusion matrix contained 82 true negatives, 8 false positives, 13 false negatives, and 77 true positives. Sensitivity was 0.856, specificity was 0.911, and precision was 0.906. The Brier score was 0.102 (95% CI, 0.076–0.132). The calibration intercept was 0.109 and the slope was 1.164, indicating modest mean underprediction with a slope close to one, although calibration estimates remain uncertain in this sample. Complete estimates for the frozen primary model are presented in Table 3.

**Figure 2:**
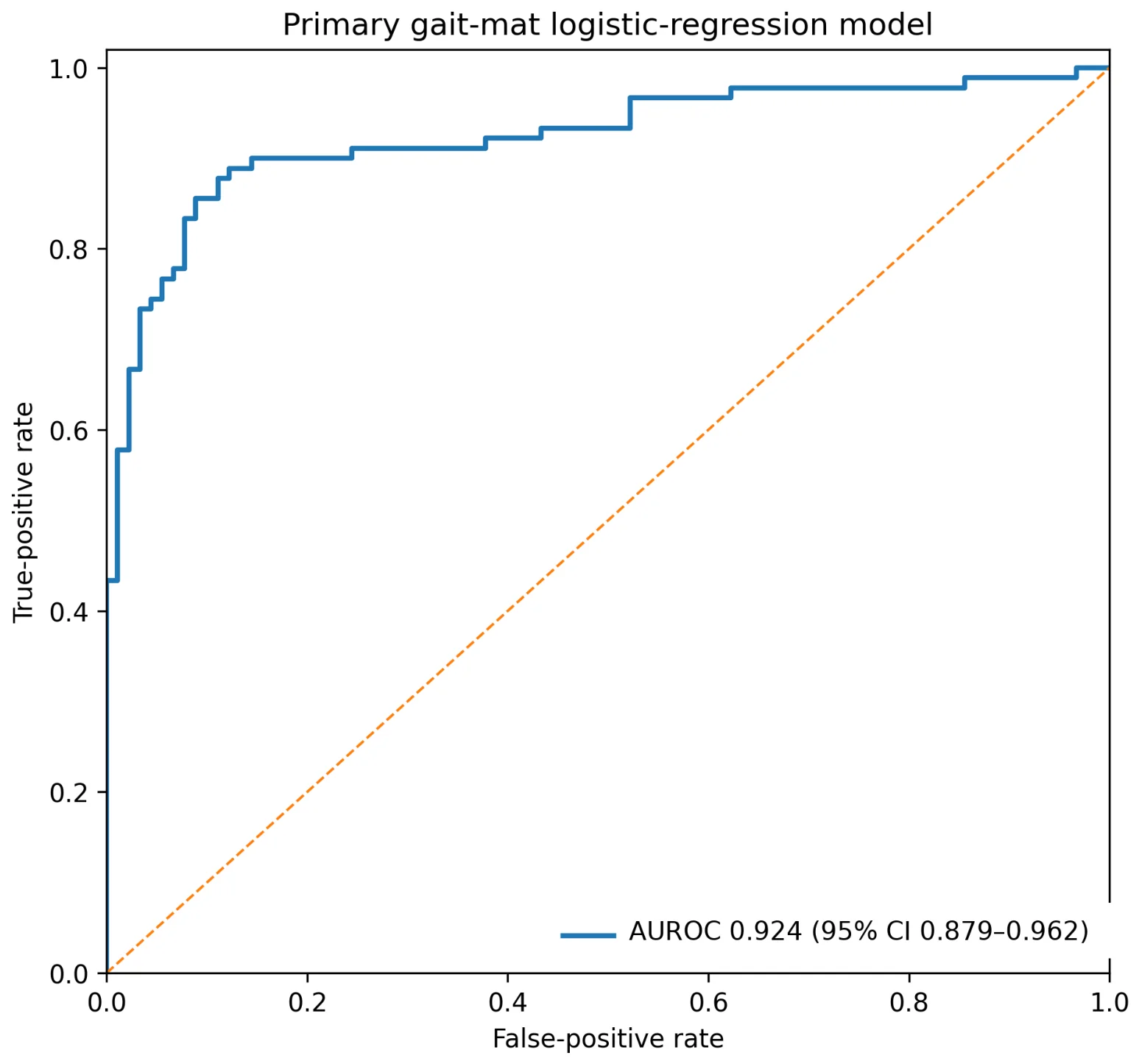
Receiver operating characteristic curve for the frozen primary gait mat logistic regression model based on mean repeated out-of-fold probabilities. The dashed diagonal line denotes chance-level discrimination.

The receiver operating characteristic curve for the frozen primary model is shown in Figure 2.

**Figure 3:**
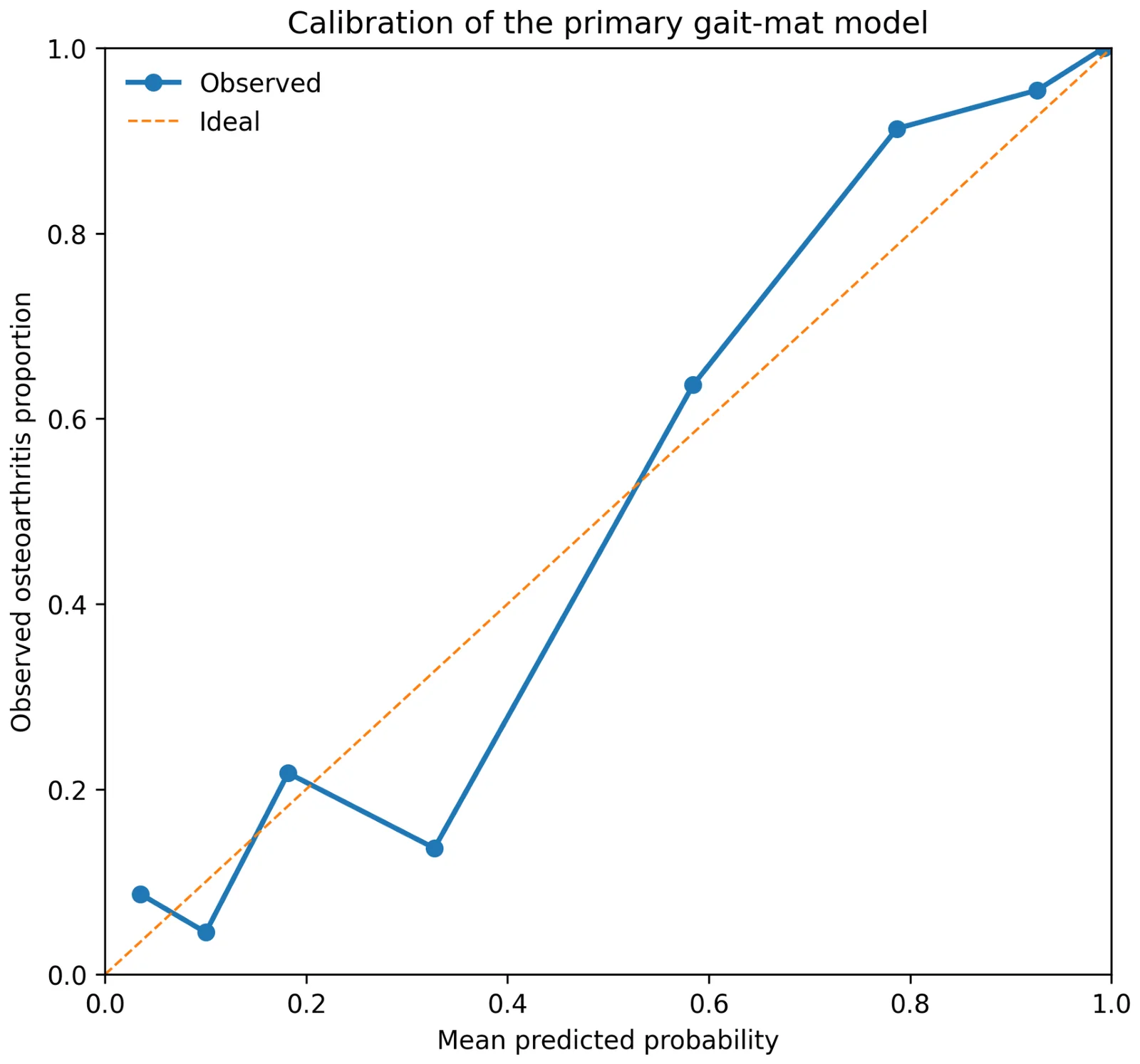
Calibration plot for the frozen primary gait mat model. Points represent eight quantile-based groups of mean repeated out-of-fold probabilities; the dashed line denotes ideal calibration. The plot is descriptive because of the limited sample size.

Calibration of the frozen primary model is shown in Figure 3.

### 3.3. Secondary Single- and Multisensor Models

Gait-mat-containing models consistently outperformed smart-insole-only and IMU-only models. The gait mat plus smart insole, gait mat plus IMU, and all-sensor models had AUROCs of 0.921, 0.917, and 0.920, respectively, compared with 0.924 for the gait mat model. The paired AUROC differences versus the primary model were −0.002 (95% CI, −0.017 to 0.012), −0.007 (−0.018 to 0.003), and −0.003 (−0.018 to 0.011), respectively. Thus, none of the added-sensor configurations demonstrated incremental discrimination in the available cohort.

The smart-insole-only and IMU-only models each had AUROC approximately 0.72 and balanced accuracy approximately 0.65. Excluding the two participants involved in the opposing-label insole duplicate did not materially change the insole plus gait mat or all-sensor AUROC (0.930 and 0.929, respectively), supporting the conclusion that the duplicate did not explain the strong gait mat-containing results. Performance estimates for all fixed sensor configurations are presented in Table 4.

**Figure 4:**
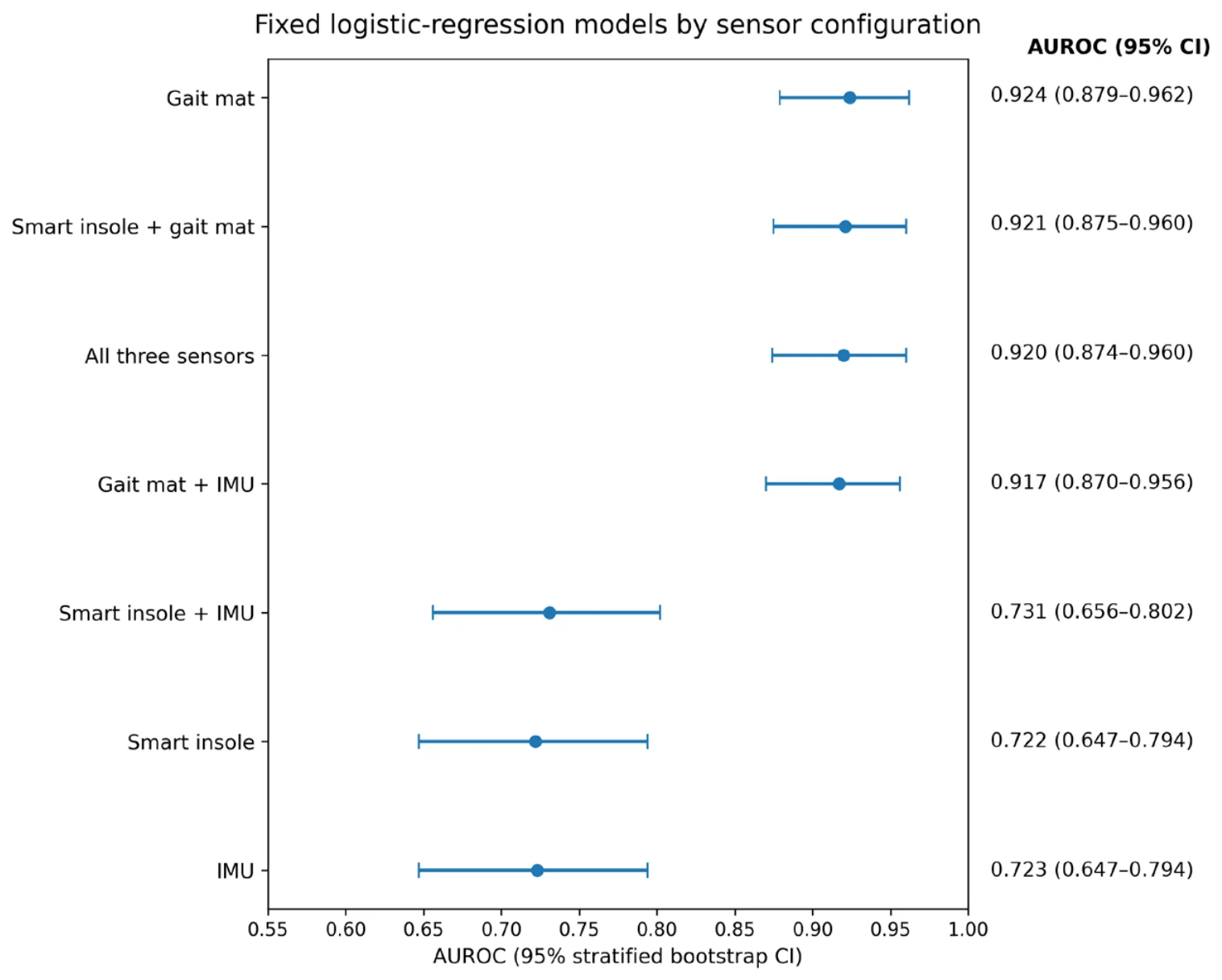
AUROC comparison across fixed logistic regression sensor configurations. The gait-mat-only model was the prespecified primary analysis. Confidence intervals are based on 10,000 outcome-stratified bootstrap resamples of participant-level mean out-of-fold predictions. Exact AUROC (95% CI) values are displayed alongside each sensor configuration.

The AUROC comparison across the fixed sensor configurations is shown in Figure 4.

### 3.4. Provider-Model Reproduction

On the 20-participant public Validation partition, the provider-supplied smart insole, smart insole plus IMU, and smart insole plus gait mat models yielded AUROCs of 0.720, 0.840, and 0.950, respectively (Table 5). The smart insole plus gait mat model classified 19 of 20 participants correctly. However, these results are descriptive because the same Validation partition was used in the provider code for early stopping and best-weight selection. The unreleased Test partition prevented independent reproduction of the provider-reported test performance.

## 4. Discussion

### 4.1. Principal Bioengineering Findings

In this public Korean knee osteoarthritis gait dataset, a compact model using nine standardized gait mat variables showed strong internal discrimination and balanced classification. Compared with the gait-mat-only AUROC of 0.924, AUROCs were 0.921 for smart insole plus gait mat, 0.917 for gait mat plus IMU, and 0.920 for all three sensors. The paired AUROC differences versus gait mat alone were −0.002 (95% CI, −0.017 to 0.012), −0.007 (−0.018 to 0.003), and −0.003 (−0.018 to 0.011), respectively, providing no evidence of incremental discrimination in this release. From a bioengineering system design perspective, the central result is that a lower-complexity instrumented gait mat representation preserved most of the discriminatory information available in this release despite the availability of additional wearable and inertial modalities. The data audit further showed that numeric sensor values and subject assignments were largely coherent while identifying metadata defects and an opposing-label duplicate that would be missed by a conventional model-only study.

The main finding is therefore not that a clinically deployable diagnostic model has been established, nor that gait mats are universally superior to wearable sensing. Rather, under a transparent subject-level internal evaluation, the gait mat offered the best trade-off observed here between biomechanical information, interpretability, and sensing/data complexity. This distinction is important because the public cohort was balanced by design, lacked an independent Test partition, and was collected within one project. Accordingly, the reported performance should be interpreted as internal evidence only and not as transportable clinical performance.

### 4.2. Biomechanical Information Content of the Gait Mat

Velocity, step length, stance percentage, swing percentage, and double-support time summarize fundamental gait adaptations associated with pain, reduced mobility, altered loading, and compensatory walking strategies. Prior work has identified spatiotemporal and kinematic gait features associated with knee osteoarthritis and radiographic severity [2,3,4]. Instrumented walkways also provide highly repeatable measurements under controlled conditions [5,6]. In engineering terms, these variables are low-dimensional, standardized summaries of bilateral timing and progression that directly reflect locomotor function. Their combination may therefore concentrate disease-related information more efficiently than a higher-dimensional representation whose features are more sensitive to daily context or preprocessing. These properties may explain why nine gait mat variables produced better performance than the much larger smart insole representation in this dataset.

### 4.3. Implications for Multisensor Bioengineering Design

The lack of multisensor gain should not be interpreted as evidence that wearables or IMUs are generally unhelpful. Wearable systems can capture real-world variability and longitudinal change, and prior studies have shown useful discrimination in independent or clinically characterized cohorts [7,8]. In the present release, however, the gait mat measured standardized spatiotemporal variables during a controlled protocol, whereas the smart insole files contained daily aggregated summaries rather than raw pressure time series, step-level waveforms, or synchronized bilateral trajectories. The IMU representation was limited to eight summary features and did not include raw accelerometer or gyroscope sequences, sensor orientation information, or synchronized joint-level waveforms. Consequently, fusion increased feature dimensionality, preprocessing burden, and potential redundancy without increasing participant count or preserving the richer temporal information that could make multimodal sensing complementary. For bioengineering design, the result illustrates that adding sensors should be justified by measurable incremental information rather than sensor count alone. Future multimodal systems should prioritize synchronized raw signals, device calibration metadata, activity context, longitudinal outcomes, and external cohorts so that sensor fusion can exploit complementary physiological and biomechanical information.

### 4.4. Data Integrity and Reproducible Bioengineering

The audit demonstrates why public medical AI datasets require file-level verification before modeling. Fourteen source-path errors did not alter the embedded sensor values, but they could break automated provenance tracing or lead users to pair a label with an incorrect file. The exact duplicate smart insole payload assigned to opposite classes is more consequential because it creates irreducible contradiction for models using those measurements. Although sensitivity analyses suggested minimal impact on gait-mat-containing models, such defects should be corrected in future releases and documented through versioned change logs.

Reproducibility was also limited by the mismatch between the documented data split and the public files. The provider code expected a Test directory, while the public release omitted that partition. Moreover, the pretrained weights were selected using the public Validation partition. Reporting a high validation accuracy without this context could be mistaken for independent test performance. Separating tuning data from evaluation data and releasing preprocessing objects, feature schemas, random seeds, and complete partitions would materially strengthen future benchmark use.

### 4.5. Translational Bioengineering Interpretation

The primary model may be useful as a reproducible bioengineering benchmark for comparing gait-measurement and feature extraction pipelines. Its relatively low Brier score and near-unit calibration slope are encouraging, but calibration must be re-estimated in the target setting before predicted probabilities are used. From a translational engineering perspective, a gait-mat-only configuration could reduce wearable device burden, sensor synchronization, calibration, and data management requirements if comparable performance were confirmed externally. However, the 0.50 decision threshold was selected for methodological consistency, not because it reflects the clinical consequences of missed or false-positive screening. Decision curve analysis was not performed because clinically justified threshold probabilities and downstream action pathways were unavailable.

The cohort was intentionally balanced at 50% prevalence, whereas knee osteoarthritis prevalence and case mix vary by age, care setting, symptom status, and diagnostic criteria. Consequently, precision and predicted probabilities cannot be directly transported to screening populations. A translational bioengineering evaluation should prespecify the intended use, recruit a representative consecutive cohort, apply an independent reference standard, and assess discrimination, calibration, net benefit, acquisition time, sensor burden, failure modes, and operational cost across clinically relevant thresholds [12,13,14,15].

### 4.6. Strengths and Limitations

Strengths include complete parsing of all public JSON files, exact raw-label value checks, subject-level cross-sensor linkage, prevention of repeated-measure leakage, a frozen final model and threshold, repeated cross-validation, outcome-stratified bootstrap intervals, paired sensor-fusion comparisons, calibration assessment, and direct inspection of provider source code and weights.

Several limitations require emphasis. First, no independent public Test data were available; all main estimates are internal and should not be interpreted as transportable clinical performance. Second, the final plan was frozen after preliminary exploratory model comparisons, so the intervals do not incorporate uncertainty from selecting the gait mat model and may be optimistic relative to a fully prospective locked analysis. Third, the bootstrap resampled frozen participant-level predictions rather than repeating the entire cross-validation and model-selection process; the intervals therefore quantify conditional uncertainty around the frozen predictions rather than the full variability of model development. Fourth, the sample was modest and balanced by design, and the osteoarthritis group was older and more often female; because gait is influenced by age, sex, body size, and mobility, residual demographic confounding cannot be excluded even though demographics were not model inputs. Fifth, the outcome was a provider class label without radiographic grade, symptom scale, severity, treatment history, or independent re-adjudication, which limits the clinical interpretation of the classification target. Sixth, the dataset came from one national project and one principal clinical institution, limiting geographic and procedural generalizability. Finally, the available features were aggregated sensor summaries rather than raw synchronized signals, so we could not evaluate temporal morphology, sensor drift, synchronization error, hardware calibration effects, or alternative sequence-level fusion architectures.

## 5. Conclusions

A subject-level data integrity audit and leakage-resistant bioengineering evaluation of a public knee osteoarthritis gait dataset showed that a fixed nine-variable gait mat logistic regression model achieved AUROC 0.924 and balanced accuracy 0.883. Compared with the gait-mat-only model, AUROCs were 0.921 for smart insole plus gait mat, 0.917 for gait mat plus IMU, and 0.920 for all three sensors, with paired AUROC differences of −0.002 (95% CI, −0.017 to 0.012), −0.007 (−0.018 to 0.003), and −0.003 (−0.018 to 0.011), respectively. Thus, smart insole and IMU additions did not improve discrimination in this release despite increasing sensing and feature complexity. The release was numerically coherent overall but contained source-path metadata errors, one opposing-label insole duplicate, and no public Test partition. In this dataset, standardized gait mat features offered the most favorable observed balance between biomechanical information, interpretability, and system simplicity. These findings support the gait mat model as a reproducible internal bioengineering benchmark, not as a validated clinical diagnostic tool. Independent, prospective, prevalence-representative evaluation using synchronized raw signals and a clearly defined reference standard is required before clinical translation.

## Figures and Tables

**Table 1:** Participant characteristics in the publicly released cohort.

Characteristic	Normal	Knee OA	Total
Participants, *n*	90	90	180
Public Training/Validation, *n*	80/10	80/10	160/20
Female, *n* (%)	60 (66.7)	74 (82.2)	134 (74.4)
Age, years	50.0 ± 8.7	58.7 ± 9.7	54.3 ± 10.1
Height, cm	165.3 ± 8.3	160.6 ± 7.8	162.9 ± 8.4
Weight, kg	63.5 ± 13.7	63.2 ± 12.7	63.3 ± 13.2
Body mass index, kg/m^2^	23.0 ± 3.4	24.4 ± 3.7	23.7 ± 3.6
OA side: left/right/bilateral, *n*	Not applicable	8/12/70	8/12/70

*Note:* Continuous variables are mean ± standard deviation. Demographic comparisons are descriptive; no between-group hypothesis tests were prespecified. OA, osteoarthritis. Age and sex differences between groups reflect the baseline characteristics of the public release.

**Table 2:** Summary of the public-data integrity audit.

Audit Item	Result	Interpretation
JSON files parsed	1080/1080	No failures
Unique public participants	180	90 normal; 90 knee OA
Training-Validation subject overlap	0	Subject separation preserved
Cross-sensor participant/class inconsistency	0	All three sensors aligned
Required-key or schema errors	0	All expected structures present
Raw-label sensor value mismatches	0	Exact value agreement
Range violations	0	Against documented bounds
Smart insole day count errors	0	All subjects had day_1-day_10
Source-path metadata errors	14	11 gait mat; 3 IMU
Opposing-label exact duplicate payloads	1 pair	Smart insole: SUBJ_247/SUBJ_253

*Note:* OA, osteoarthritis; IMU, inertial measurement unit. The 14 source-path defects did not alter the paired sensor values used in analysis.

**Table 3:** Performance of the frozen primary gait mat logistic regression model.

Measure	Estimate	95% Stratified Bootstrap CI
AUROC	0.924	0.879–0.962
AUPRC	0.940	0.905–0.969
Balanced accuracy	0.883	0.833–0.928
Accuracy	0.883	0.833–0.928
F1 score	0.880	0.827–0.927
Sensitivity	0.856	0.778–0.922
Specificity	0.911	0.844–0.967
Precision	0.906	0.848–0.963
Brier score	0.102	0.076–0.132
Calibration intercept	0.109	Descriptive
Calibration slope	1.164	Descriptive

*Note:* Performance was estimated from each participant’s mean of 10 repeated out-of-fold probabilities. Confidence intervals were derived from 10,000 outcome-stratified bootstrap resamples for discrimination, classification, and Brier score measures; calibration intercept and slope are descriptive point estimates. AUROC, area under the receiver operating characteristic curve; AUPRC, area under the precision recall curve.

**Table 4:** Fixed logistic regression models by sensor configuration.

Predictor Set	AUROC (95% CI)	Balanced Accuracy (95% CI)	Brier Score (95% CI)
Gait mat	0.924 (0.879–0.962)	0.883 (0.833–0.928)	0.102 (0.076–0.132)
Smart insole	0.722 (0.647–0.794)	0.650 (0.578–0.717)	0.215 (0.187–0.244)
IMU	0.723 (0.647–0.794)	0.644 (0.572–0.711)	0.209 (0.184–0.235)
Smart insole + gait mat	0.921 (0.875–0.960)	0.872 (0.822–0.917)	0.105 (0.078–0.135)
Smart insole + IMU	0.731 (0.656–0.802)	0.661 (0.594–0.728)	0.210 (0.182–0.239)
Gait mat + IMU	0.917 (0.870–0.956)	0.856 (0.800–0.906)	0.108 (0.080–0.139)
All three sensors	0.920 (0.874–0.960)	0.867 (0.817–0.917)	0.105 (0.078–0.136)

*Note:* All models used the same fixed logistic regression specification and subject-level repeated cross-validation. Lower Brier scores are better. CI, outcome-stratified percentile bootstrap confidence interval.

**Table 5:** Descriptive reproduction of provider-supplied pretrained models on the public Validation partition (*n* = 20).

Provider Model	AUROC (95% CI)	Balanced Accuracy (95% CI)	Sensitivity	Specificity	Brier
Smart insole	0.720 (0.460–0.920)	0.650 (0.450–0.850)	0.700	0.600	0.215
Smart insole + IMU	0.840 (0.630–1.000)	0.800 (0.600–0.950)	0.800	0.800	0.159
Smart insole + gait mat	0.950 (0.820–1.000)	0.950 (0.850–1.000)	0.900	1.000	0.064

*Note:* These intervals reflect only the 20-participant public Validation partition. They are not independent test estimates because provider model selection used this partition. IMU, inertial measurement unit.

## Data Availability

Restrictions apply to the availability of the source data. The data analyzed in this study were obtained from the AI Hub “Musculoskeletal Disease Biosignal Data” dataset (dataset no. 71966). The source data are accessible through AI Hub subject to user registration, data use approval, and compliance with the applicable terms of use. The original source files are not included in this article or its Supplementary Materials. The frozen analysis plan, analysis code, data audit outputs, derived predictions, aggregate results, and supporting tables generated in this study are provided in the accompanying Supplementary Materials. Inquiries regarding access to the original source data should be directed to AI Hub.

## Supplementary Materials

The following supporting information can be downloaded at: https://www.mdpi.com/article/10.3390/bioengineering13090965/s1, The supplementary package contains the frozen analysis plan, detailed audit outputs, complete performance tables, paired AUROC differences, provider-model reproduction results, and executable analysis scripts. Figure source files are supplied separately at 300 dpi.

## Abbreviations

Abbreviations used in this manuscript are listed below.

Abbreviation	Definition
AUPRC	Area under the precision-recall curve
AUROC	Area under the receiver operating characteristic curve
CI	Confidence interval
IMU	Inertial measurement unit
OA	Osteoarthritis
OOF	Out-of-fold
TRIPOD + AI	Transparent Reporting of a multivariable prediction model for Individual Prognosis Or Diagnosis plus Artificial Intelligence
