# Supplementary material for: Leakage-Resistant Evaluation of Gait Mat and Multisensor Biomechanical Features for Knee Osteoarthritis Screening: A Subject-Level Data Integrity Study
Source: Bioengineering (Basel). 2026 Aug 24;13(9):965. doi: 10.3390/bioengineering13090965 (PMC13603537; doi:10.3390/bioengineering13090965)
Supplement: Supplementary file 1 [file bioengineering-13-00965-s001.zip › Supplementary Materials.pdf]

Supplementary Materials

Leakage-Resistant Evaluation of Gait-Mat and Multisensor Biomechanical Features for Knee Osteoarthritis Screening: A Subject-Level Data Integrity Study

Mi-Ae Yang and Kang-Su Ha

Supplementary File S1. Frozen Final Analysis Plan

FINAL LOCKED ANALYSIS PLAN

Freeze date: 2026-07-31

Random seed: 20260731

Population: 180 publicly released subjects (90 normal, 90 knee osteoarthritis), combining public Training and Validation partitions because the documented Test partition was not released.

Unit of analysis and resampling: subject.

Primary predictors: all 9 gait-mat variables.

Primary model: class-balanced L2 logistic regression, C=0.3, liblinear solver, 0.5 decision threshold.

Preprocessing: median imputation and standardization estimated within each training fold.

Internal evaluation: repeated stratified 5-fold cross-validation, 10 repeats. Each subject contributes one out-of-fold probability per repeat; probabilities are averaged across repeats before performance estimation.

Primary metrics: AUROC and balanced accuracy. Secondary metrics: AUPRC, accuracy, F1, sensitivity, specificity, precision, Brier score, calibration intercept, calibration slope.

Uncertainty: 10,000 nonparametric bootstrap resamples stratified by outcome class, resampling subject-level mean out-of-fold predictions; percentile 95% confidence intervals.

Secondary analyses: fixed logistic-regression models for single- and multisensor configurations; provider-supplied pretrained model reproduction on the public Validation partition; duplicate-exclusion sensitivity analysis.

No external validation is claimed. The plan was not prospectively registered; it was frozen before final bootstrap confidence-interval estimation and manuscript-level interpretation.

Supplementary Table S1. Full Fixed-Model Performance

Predictor set	AUROC (95% CI)	AUPRC (95% CI)	Balanced accuracy (95% CI)	Sensitivity	Specificity	Precision	Brier (95% CI)	Cal. intercept	Cal. slope
Gait mat	0.924 (0.879-0.962)	0.940 (0.905-0.969)	0.883 (0.833-0.928)	0.856	0.911	0.906	0.102 (0.076-0.132)	0.109	1.164
Smart insole	0.722 (0.647-0.794)	0.749 (0.680-0.817)	0.650 (0.578-0.717)	0.622	0.678	0.659	0.215 (0.187-0.244)	0.045	0.759
IMU	0.723 (0.647-0.794)	0.766 (0.702-0.829)	0.644 (0.572-0.711)	0.611	0.678	0.655	0.209 (0.184-0.235)	0.010	0.893
Smart insole + gait mat	0.921 (0.875-0.960)	0.939 (0.905-0.968)	0.872 (0.822-0.917)	0.844	0.900	0.894	0.105 (0.078-0.135)	0.112	1.015
Smart insole + IMU	0.731 (0.656-0.802)	0.754 (0.682-0.825)	0.661 (0.594-0.728)	0.611	0.711	0.679	0.210 (0.182-0.239)	0.060	0.789
Gait mat + IMU	0.917 (0.870-0.956)	0.935 (0.901-0.965)	0.856 (0.800-0.906)	0.833	0.878	0.872	0.108 (0.080-0.139)	0.085	1.044
All sensors	0.920 (0.874-0.960)	0.938 (0.904-0.967)	0.867 (0.817-0.917)	0.833	0.900	0.893	0.105 (0.078-0.136)	0.115	1.014

Note: CIs were derived from 10,000 outcome-stratified resamples of participant-level mean out-of-fold predictions. Calibration intercept and slope are descriptive point estimates.

Supplementary Table S2. Paired AUROC Differences versus the Primary Gait-Mat Model

Comparator	AUROC difference	95% paired bootstrap CI	Resamples
Smart insole + gait mat	-0.002	-0.017 to 0.012	10,000
Gait mat + IMU	-0.007	-0.018 to 0.003	10,000
All sensors	-0.003	-0.018 to 0.011	10,000

Supplementary Table S3. Provider-Model Reproduction on the Public Validation Partition

Provider model	n	AUROC (95% CI)	AUPRC (95% CI)	Balanced accuracy (95% CI)	F1	Brier (95% CI)	TN/FP/FN/TP
Smart insole	20	0.720 (0.460-0.920)	0.757 (0.571-0.934)	0.650 (0.450-0.850)	0.667	0.215 (0.143-0.293)	6/4/3/7
Smart insole + IMU	20	0.840 (0.630-1.000)	0.812 (0.627-1.000)	0.800 (0.600-0.950)	0.800	0.159 (0.087-0.244)	8/2/2/8
Smart insole + gait mat	20	0.950 (0.820-1.000)	0.967 (0.887-1.000)	0.950 (0.850-1.000)	0.947	0.064 (0.010-0.152)	10/0/1/9

Note: The public Validation partition was used by the provider code for early stopping and best-weight selection; these are descriptive reproduction results, not independent test estimates.

Supplementary Table S4. Detailed Source-Path Metadata Defects

Partition	Sensor	Label file	PID	Class	Expected raw file	Reason
train	Gait mat	lb_01_pad_084.json	SUBJ_084	0	01_pad_084.json	source folder class differs from class value
train	Gait mat	lb_01_pad_086.json	SUBJ_086	0	01_pad_086.json	source folder class differs from class value
train	Gait mat	lb_01_pad_088.json	SUBJ_088	0	01_pad_088.json	source basename differs from label-derived raw filename; source folder class differs from class value
train	Gait mat	lb_01_pad_093.json	SUBJ_093	0	01_pad_093.json	source folder class differs from class value
train	Gait mat	lb_01_pad_094.json	SUBJ_094	0	01_pad_094.json	source folder class differs from class value
train	Gait mat	lb_01_pad_095.json	SUBJ_095	0	01_pad_095.json	source folder class differs from class value
train	Gait mat	lb_01_pad_096.json	SUBJ_096	0	01_pad_096.json	source folder class differs from class value
train	Gait mat	lb_01_pad_097.json	SUBJ_097	0	01_pad_097.json	source folder class differs from class value
train	Gait mat	lb_01_pad_098.json	SUBJ_098	0	01_pad_098.json	source folder class differs from class value
train	Gait mat	lb_01_pad_107.json	SUBJ_107	0	01_pad_107.json	source folder class differs from class value
train	Gait mat	lb_02_pad_083.json	SUBJ_083	1	02_pad_083.json	source basename differs from label-derived raw filename
train	IMU sensor	lb_01_imu_272.json	SUBJ_272	0	01_imu_272.json	source basename differs from label-derived raw filename
train	IMU sensor	lb_01_imu_274.json	SUBJ_274	0	01_imu_274.json	source basename differs from label-derived raw filename
train	IMU sensor	lb_02_imu_202.json	SUBJ_202	1	02_imu_202.json	source folder class differs from class value

Supplementary Table S5. Exact Duplicate Sensor Payload with Conflicting Labels

Partition	Sensor	Records	Class conflict	Files
train	Smart insole	2	YES	lb_01_insole_247.json (PID=SUBJ_247, class=0) | lb_02_insole_253.json (PID=SUBJ_253, class=1)

Supplementary Table S6. Duplicate-Exclusion Sensitivity Analysis

Predictor set	n	AUROC	Balanced acc.	Accuracy	F1	Sensitivity	Specificity	Brier
Smart insole + gait mat	178	0.930	0.871	0.871	0.869	0.854	0.888	0.100
All sensors	178	0.929	0.871	0.871	0.869	0.854	0.888	0.100
Smart insole + IMU	178	0.762	0.713	0.713	0.702	0.674	0.753	0.198
Smart insole	178	0.741	0.697	0.697	0.703	0.719	0.674	0.205

Note: SUBJ_247 and SUBJ_253 were excluded because their smart-insole sensor payloads were exactly identical but their labels conflicted.
